# Safe, accurate, and precise sulfur isotope analyses of arsenides, sulfarsenides, and arsenic and mercury sulfides by conversion to barium sulfate before EA/IRMS

**DOI:** 10.1007/s00216-021-03854-y

**Published:** 2022-01-23

**Authors:** Jorge E. Spangenberg, Nicolas J. Saintilan, Sabina Strmić Palinkaš

**Affiliations:** 1 Institute of Earth Surface Dynamics (IDYST), University of Lausanne, 1015 Lausanne, Switzerland; 2 Institute of Geochemistry and Petrology, Department of Earth Sciences, ETH, 8092 Zürich, Switzerland; 3 Department of Geosciences, UiT The Arctic University of Norway in Tromsø, 9037 Tromsø, Norway

**Keywords:** EA combustions, Barium chloride, Hydrogen peroxide, Realgar, Cinnabar, Orpiment, Arsenopyrite, Sulfite oxidation

## Abstract

The stable isotope ratios of sulfur (*δ*^34^S relative to Vienna Cañon Diablo Troilite) in sulfates and sulfides determined by elemental analysis and isotope ratio mass spectrometry (EA/IRMS) have been proven to be a remarkable tool for studies of the (bio)geochemical sulfur cycles in modern and ancient environments. However, the use of EA/IRMS to measure δ^34^S in arsenides and sulfarsenides may not be straightforward. This difficulty can lead to potential health and environmental hazards in the workplace and analytical problems such as instrument contamination, memory effects, and a non-matrix-matched standardization of δ^34^S measurements with suitable reference materials. To overcome these practical and analytical challenges, we developed a procedure for sulfur isotope analysis of arsenides, which can also be safely used for EA/IRMS analysis of arsenic sulfides (i.e., realgar, orpiment, arsenopyrite, and arsenian pyrite), and mercury sulfides (cinnabar). The sulfur dioxide produced from off-line EA combustion was trapped in an aqueous barium chloride solution in a leak-free system and precipitated as barium sulfate after quantitative oxidation of hydrogen sulfite by hydrogen peroxide. The derived barium sulfate was analyzed by conventional EA/IRMS, which bracketed the δ^34^S values of the samples with three international sulfate reference materials. The protocol (BaSO_4_-EA/IRMS) was validated by analyses of reference materials and laboratory standards of sulfate and sulfides and achieved accuracy and precision comparable with those of direct EA/IRMS. The δ^34^S values determined by BaSO_4_-EA/IRMS in sulfides (arsenopyrite, arsenic, and mercury sulfides) samples from different origins were comparable to those obtained by EA/IRMS, and no sulfur isotope fractionations were introduced during sample preparation. We report the first sulfur isotope data of arsenides obtained by BaSO_4_-EA/IRMS.

**Graphical abstract:**

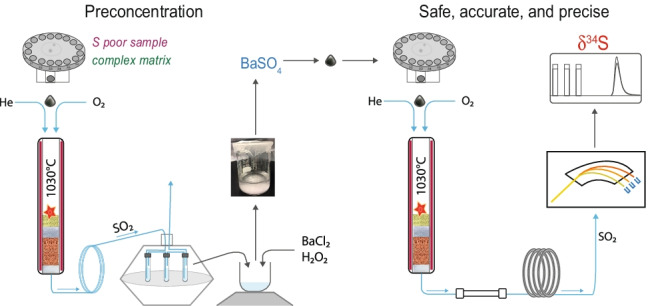

**Supplementary Information:**

The online version contains supplementary material available at 10.1007/s00216-021-03854-y.

## Introduction

The sulfur stable isotopes (δ^34^S) in sulfates and sulfides have been proven to be a remarkable tool for studying geochemical and biogeochemical cycles in modern and ancient environments [1–3]. Particularly, in magmatic-hydrothermal and hydrothermal systems and the associated ore deposits, the δ^34^S values of sulfides routinely provide constraints on the source of sulfur (e.g., magmatic and biogenic) and the processes (e.g., sulfate reduction, fluid mixing, and water–rock interactions) and environmental parameters (e.g., temperature, oxygen fugacity, and pH) associated with sulfide precipitation [4–6]. In some peculiar magmatic-hydrothermal and hydrothermal mineral deposits, the mineralogy may be dominated by arsenides, sulfarsenides, and arsenic (As) and mercury (Hg) sulfides. Arsenic is bonded to sulfur in realgar (> 90% arsenic disulfide, As_2_S_2_), orpiment (arsenic trisulfide, As_2_S_3_), intermediate As-S compounds (As_1−x_S_x_), sulfarsenides with the pyrite-structure (MAs_x_S_2−x_, with M representing a metal such Fe, Co, Ni, Cu, Pb, Zn, Au, Ag, and platinum group elements, and x < 1), and also sulfur-containing arsenides [7]. The most common sulfarsenides in hydrothermal systems are arsenopyrite (ferrous arsenic sulfide, FeAsS) and arsenian pyrite (FeAs_x_S_2−x_ with x < 1), together with cobaltite (CoAsS), enargite (Cu_3_AsS_4_), gersdorffite (NiAsS), and glaucodot ((Co,Fe)AsS) [7, 8]. These sulfarsenides are often found together with cobalt–nickel mono-, di-, and tri-arsenides, which may contain up to 3 wt.% sulfur [9, 10]. Furthermore, and importantly, arsenic is a ubiquitous environmental toxicant that adversely impacts human health (i.e., induces cancer, DNA hypomethylation, and arsenicosis) and ecosystem health (i.e., causes pollution) [11, 12]. Arsenic disulfide is highly toxic, which restricts its medical applications [11].

Mercury sulfide (HgS) is the most abundant form of mercury in nature, particularly in the two crystal forms of cinnabar (*α*-HgS, hexagonal, red) and metacinnabar (*β*-HgS, cubic, black). Sedimentary and volcanic rocks host HgS deposits in the lithosphere along convergent boundaries in recent and ancient mountain belts [13]. The mining of these deposits initiated anthropogenic cycling of mercury [14]. Sulfur isotope analyses of HgS samples from the large mercury mines of Almadén (Spain) [15] and Idrija (Slovenia) [16] revealed multiple sources of sulfur (e.g., sedimentary and magmatic), constraining the role of hydrothermal systems triggered by volcanic activity in the origin of mercury mineralization. Furthermore, the net formation (precipitation vs. dissolution) of HgS is one of the major mercury sinks in the environment, as it removes this element from biogeochemical and anthropogenic cycling [17]. Through these cycles, HgS may form nanometer- to micrometer-sized particles in a wide range of matrices (e.g., mine waste, coal, gypsum, airborne particles, soils and sediments, and biological materials) [13, 18]. Due to the paramount importance of controlling the biogeochemical cycle of mercury by HgS [18], the accurate and precise analysis of sulfur isotopes could provide promising information for the characterization of the HgS phases (*α*-HgS, *β*-HgS) in different matrices and the study of their formation, dissolution, and translocation between environmental compartments. However, to the best of our knowledge, there are no published sulfur isotope data of fine-grained HgS in environmental samples.

Sulfur stable isotope ratios are commonly obtained by elemental analysis (EA) coupled with isotope ratio mass spectrometry (IRMS) [19, 20]. The EA/IRMS system automates the combustion, drying, and chromatographic purification of the produced sulfur oxide (SO_2_) before it enters the ion source of the mass spectrometer for sulfur isotope analysis. The measurement of sulfur isotopes in sulfur-containing arsenides, as well as arsenic and mercury sulfides, by EA/IRMS can be problematic. Improper storage, handling, and analytical protocols for the analysis of arsenic- and mercury-containing samples can lead to potential health hazards at the workplace and mass spectrometric and analytical problems (e.g., instrument contamination and memory effects). The difficulty is mainly rooted in the low sulfur content of the analyte (i.e., arsenides with less than 1.0 wt.% total sulfur, TS), because the acquisition of a workable and reliable signal (i.e., the integrated area of the *m*/*z* 64 and 66 peaks higher than 10 V seconds, Vs) relies on the combustion of large aliquots () in the elemental analyzer. The use of such large aliquots has the detrimental effect of potentially introducing and accumulating a relatively high amount of other non-analyte gases in the ion source (i.e., noxious and corrosive arsenic or mercury gases in this case). This matrix effect allows residual gases and atoms of previous samples into the different components of the EA/IRMS system (reactor, water trap, GC-column, capillaries, source). These gases, which may include the toxic arsenic and mercury gases in this case, may cause instrument contamination and contribute to the memory effect caused by “sticky” SO_2_ gas as well as peak tailing and high background problems, all of which will compromise the accuracy and precision of the δ^34^S results. Thermodynamic and experimental data on the behavior of arsenic in the Cu-Fe-S system show that during copper smelting/conversion at temperatures of 1000–1250 °C, arsenic is eliminated through oxidation into the metal oxide mixture (i.e., slag) and transferred into the gas phase mainly as arsenic oxides (e.g., AsO_2_, AsO, As_4_O_10_, As_2_O_3_, As_4_O_6_, As_4_O_7_, and As_4_O_8_) and arsenic gas (As_2_) [21–23]. Similarly, at a high temperature (> 450 °C), any mercury compound, including HgS and mercuric oxide (HgO), decomposes to form elemental mercury [24, 25]. Therefore, toxic arsenic- and mercury-containing gases may be released upon combustion in the reactor of the elemental analyzer containing tungsten oxide (WO_3_) and reduced copper at 1030 °C when used for sulfur isotope analysis. Finally, a further analytical problem may be the lack of suitable (chemical-matrix-matching) international reference materials for arsenides and arsenic and mercury sulfides. The use of well-prepared laboratory standards may alleviate this complication, but the limitation in assessing the accuracy of the measured δ^34^S values remains unresolved.

An alternative instrumental approach for precise and accurate sulfur isotope measurements in low-sulfur samples may be the use of multi-collector inductively coupled plasma mass spectrometry (MC-ICP-MS) [26, 27]. During the last decade, MC-ICP-MS methods have been developed for the determination of δ^34^S in nanomole sulfur quantities with a reproducibility better than 0.2 mUr (or ‰, see below) for 2 SD [28–30]. The small sulfur amount needed for precise δ^34^S measurement is as little as 5 nmol, which is one-hundredth the amount required (ca 1 µmol S) to achieve similarly precise sulfur isotope analysis by EA/IRMS [30]. Generally, the δ^34^S values measured by MC-ICP-MS compare well with those obtained by EA/IRMS [30]. However, some effects, including high blank to sample ratios and non-quantitative (< 95%) recovery of sulfate during the wet chemical processing (i.e., concentrated acid treatments, evaporations to dryness, dissolution, and ion-exchange chromatography for separation of matrix constituents) performed to obtain the analyte sulfate solution [26, 28, 31], can cause non-reproducible and inaccurate sulfur isotope measurements in low-sulfur samples [30].

Previous studies have shown that, using laboratory standards and commercially available sulfide reference materials for normalization of the δ^34^S values, EA/IEMS could be successfully applied to the sulfur isotope analysis of mercury sulfide samples from European mercury deposits and fine-grained *α*-HgS in archaeological red pigments [16, 32].

The primary goal of this work was to develop a method for sulfur isotope analysis in samples with low to very low sulfur content and complex matrices, probably containing hazardous (e.g., toxic and corrosive) components. In such challenging samples, the separation and preconcentration of the analyte are recommended. The proposed analytical procedure, hereafter referred to as BaSO_4_-EA/IRMS, introduces an off-line matrix separation and sulfur preconcentration step based on barium sulfate formation in the trapping solution collecting gaseous products from elemental analyzer. The sulfur dioxide (SO_2_) produced by replicate combustions of aliquots of the sulfur-containing sample is trapped in a barium chloride solution. With further oxidation of the SO_2_ to sulfur trioxide (SO_3_) by the addition of hydrogen peroxide (H_2_O_2_), barium sulfate (BaSO_4_) readily precipitates. BaSO_4_ is recovered and analyzed for sulfur isotopes by EA/IRMS using well-constrained matrix-matched reference materials (RMs) for normalization of the measured δ^34^S values. The BaSO_4_-EA/IRMS method was validated through the analysis of sulfate and sulfide RMs. The accuracy and precision of δ^34^S values in arsenic and mercury sulfides obtained by BaSO_4_-EA/IRMS are comparable with those from direct EA/IRMS, indicating that the matrix-separation/sulfur-preconcentration step did not cause sulfur isotope fractionations. Thus, we believe that the developed analytical procedure, BaSO_4_-EA/IRMS, is a promising and powerful strategy for analyzing the sulfur isotopes of a wide range of organic and inorganic samples with low to very low concentrations of sulfur and potentially challenging matrices, such as arsenides, soils and sediments, coal, petroleum, sulfur-vulcanized rubbers, foodstuffs, and many biological materials.

## Materials and methods

### Materials, chemicals, and solutions

Solutions were prepared from fuming hydrochloric acid, 37% RPE-ISO, containing $\le$ 1 mg/kg sulfate and $\le$ 10.5 mg/kg sulfite (Carlo Erba Reagents, Val de Reuil, France), barium chloride (Suprapur®, 99.995%, Merck, Darmstadt, Germany), and water with an 18.2 MΩ cm resistivity at 25 °C (MQ water) obtained from a Direct-Q UV 3 Millipore® system (Merck, Darmstadt, Germany). Hydrogen peroxide solution, with $\ge$ 30% and < 0.1 mg/kg sulfate for trace analysis (Sigma-Aldrich, Steinheim, Germany), was purchased from Merck Life Science (Buchs, Switzerland). All glassware used for handling solutions and samples in the experiments was thoroughly washed, rinsed with deionized and purified water, and heated at 480 °C for > 4 h before use. The elemental analyzer reactor comprised a transparent quartz tube with a 450 mm length, an 18 mm outer diameter (OD) and 14 mm internal diameter (ID), quartz wool, quartz chips, reduced copper wire with 0.7 mm diameter, 12–35 mesh tungsten trioxide (WO_3_) granulate, and magnesium perchlorate (Mg(ClO_4_)_2_), all purchased from Säntis Analytical (Teufen, Switzerland). Vanadium pentoxide (V_2_O_5_) was obtained from Thermo Fisher Scientific (Bremen, Germany). Helium (purity 99.999%) and oxygen (99.998%) were purchased from Air Liquide/Carbagas (Lausanne, Switzerland), and sulfur dioxide (99.98%) was purchased from Multigas (Domdidier, Switzerland). Standard laboratory ware and protective equipment (laboratory coats, masks, and gloves) were worn for the preparation of powders of arsenic and mercury minerals and when adding V_2_O_5_. The disposal of used solutions and elemental analyzer reactors followed the safety and security guidelines for hazardous waste management at UNIL laboratories.

### Samples and sample preparation

The BaSO_4_-EA/IRMS procedure was validated by analyzing international reference materials (RMs) and laboratory standards (Table 1) and compared with results obtained by direct EA/IRMS on the same material. A suite of arsenides and arsenic and mercury sulfides (Table 2) from different depositional environments and geological ages were used to optimize the BaSO_4_-EA/IRMS protocol and test the applicability of BaSO_4_ preconcentration before EA/IRMS. Five mineralized samples containing the monoarsenide nickeline (NiAs), diarsenides rammelsbergite (NiAs_2_) and safflorite (CoAs_2_), and/or the triarsenide skutterudite (CoAs_3_) were selected from a suite of samples collected in the Bou Azzer mine district (Morocco) [10, 33]. Nickeline and rammelsbergite coexist in single-hand samples, whereas safflorite and skutterudite are recognized as discrete mineralizations in different samples. Pure monophasic mineral separates were obtained at the Institute of Geochemistry and Petrology of the ETH (Zürich, Switzerland) using a procedure described previously [34]. Briefly, this workflow used the 70–200 mesh size fractions of the ground sample and combined a Frantz Isodynamic Separator (FIS; S.G. Frantz Co., Tullytown, PA, USA) and heavy liquid separation of the magnetic (M) and nonmagnetic (NM) fractions. Trace amounts of arsenopyrite and magnetite were removed by concentrating the arsenides into the NM fraction with a current of 1.1 amp. Then, when several arsenide phases coexisted in the mineral separates, each arsenide phase was carefully handpicked under a binocular microscope to obtain pure monophasic samples. These arsenide samples contained between 0.83 and 3.03 wt.% total sulfur (TS), as shown by microprobe analyses [35].

**Table 1:** International reference materials and laboratory standards for sulfur isotope analysis used in this study

Identifier	Material	Chemical formula	Theor. *TS*^a^ (wt.%)	δ^34^S_VCDT_^b^ (mUr or ‰)	Uncertainty (*n*)
NBS 127	Barite	BaSO_4_	13.74	21.12	0.22
IAEA-SO-5	Barite	BaSO_4_	13.74	0.49	0.11
IAEA-SO-6	Barite	BaSO_4_	13.74	−34.05	0.08
IAEA-S-4 Soufre de Lacq	Elemental sulfur	S	100.00	16.90	0.12
IAEA-S-1^d^	Silver sulfide	Ag_2_S	12.94	−0.3	None
IAEA-S-2	Silver sulfide	Ag_2_S	12.94	22.62	0.16
IAEA-S-3	Silver sulfide	Ag_2_S	12.94	−32.49	0.16
NBS 122 ^e^	Sphalerite	ZnS	32.83	0.18	0.14
NBS 123 ^e^	Sphalerite	ZnS	32.83	17.09	0.31
				17.44	0.10
UVA-sulfate	Synthetic barium sulfate	BaSO_4_	13.74	12.73	0.21 (8)
Fx-sulfate	Synthetic barium sulfate	BaSO_4_	13.74	17.82	0.22 (8)
UNIL-PyE	Pyrite	FeS_2_	53.45	−6.72	0.19 (28)
UNIL-Cinnabar	Synthetic mercury (II) sulfide	HgS	13.78	15.82	0.15 (4)

^a^Theoretical total sulfur content in wt.% determined from the stoichiometry

^b^Values for the international reference materials (RMs) from Brand et al. [42]. Values for the laboratory standards obtained via EA/IRMS measurements (December 2020–January 2021) and calibration with international RMs

^c^Uncertainties for the laboratory standards correspond to one standard deviation (1-sigma) of *n* measurements

^d^Primary VCDT reference with exact value defining the δ^34^S_VCDT_ scale

^e^ Discontinued, possibly non-homogeneous[42]

**Table 2:** Overview of the studied arsenides and arsenic and mercury sulfides

Lab code^a^	Identifier	Mineral^b^	Chemical formula	Origin
AR-1	MR-BAZ-04	Nickeline	NiAs	Bou Azzer Co–Ni-arsenides mineralization, Central Anti-Atlas, Morocco [10, 22]
AR-2	MR-BAZ-14	Safflorite	CoAs_2_	Bou Azzer Co–Ni-arsenides mineralization, Central Anti-Atlas, Morocco
AR-3	MR-BAZ-15	Safflorite	CoAs_2_	Bou Azzer Co–Ni-arsenides mineralization, Central Anti-Atlas, Morocco
AR-4	MR-BAZ-18	Skutterudite	CoAs_3_	Bou Azzer Co–Ni-arsenides mineralization, Central Anti-Atlas, Morocco
AR-5	MR-BAZ-19	Skutterudite	CoAs_3_	Bou Azzer Co–Ni-arsenides mineralization, Central Anti-Atlas, Morocco
Rlg-1	MK-Rlg-1	Realgar	As_4_S_4_	Allchar Au-As-Sb-Tl Carlin-type mineralization, North Macedonia [23]
Rlg-2	MK-Rlg-2	Realgar	As_4_S_4_	Allchar Au-As-Sb-Tl Carlin-type mineralization, North Macedonia
Rlg-3	USA-Rlg	Realgar	As_4_S_4_	Getchell Carlin-Type gold deposit, north-central Nevada, USA [24]
Orp-1	MK-Orp-1	Orpiment	As_2_S_3_	Allchar Au-As-Sb-Tl Carlin-type mineralization, North Macedonia [23]
Orp-2	MK-Orp-2	Orpiment	As_2_S_3_	Allchar Au-As-Sb-Tl Carlin-type mineralization, North Macedonia
Orp-3	MK-Orp-3	Orpiment	As_2_S_3_	Allchar Au-As-Sb-Tl Carlin-type mineralization, North Macedonia
Orp-4	RO-Orp	Orpiment	As_2_S_3_	Moldova Nouă-Sasca Cu-Mo ore field, Caraş-Severin, Romania [25]
Apy-1	NOR-Apy-1	Arsenopyrite	FeAsS	Kjørisfjell As-deposit, Skjomen, Narvik, Nordland, Scandinavian Caledonides, Norway
Apy-2	NOR-Apy-2	Arsenopyrite	FeAsS	Kjørisfjell As-deposit, Skjomen, Narvik, Nordland, Scandinavian Caledonides, Norway
Apy-3	NOR-Apy-3	Arsenopyrite	FeAsS	Hølonda base metal mineralization, Scandinavian Caledonides, Sør-Trøndelag, Norway
Apy-4	BULG-Apy-1	Arsenopyrite	FeAsS	Coarse-grained arsenopyrite from the Pb–Zn mineralization, Osogovo Mts., Bulgaria
Apy-5	BULG-Apy-2	Arsenopyrite	FeAsS	Fined grained arsenopyrite from the Pb–Zn mineralization, Osogovo Mts., Bulgaria
Cin-1	SLO-ID-Cin-2	Cinnabar	HgS	Pure *α*-HgS from cinnabar of the Idrija mercury mine, NW External Dinarides, Slovenia [16]
Cin-2	SLO-ID-Cin-4b	Cinnabar	HgS	Pure *α*-HgS from cinnabar of the Idrija mercury mine, NW External Dinarides, Slovenia
Cin-3	SLO-ID-Cin-171	Cinnabar ore	HgS	Idrija mercury deposit, NW External Dinarides, Slovenia
Cin-4	ES-ALM-Cin-1	Cinnabar ore	HgS	Almadén deposit, Almadén mining district, Ciudad Real, Spain [15]
Cin-5	ES-ENT-Cin-1	Cinnabar ore	HgS	El Entredicho deposit, Almadén mining district, Ciudad Real, Spain [15, 19]

^a^AR, arsenide; Rlg, realgar; Orp, orpiment; Apy, arsenopyrite; Cin, cinnabar

^b^Samples are pure mineral separates, except the cinnabar ore samples from Almadén (fine-grained cinnabar in quartzite) and Idrija (fine-grained cinnabar in Lower Scythian dolostone)

Realgar, orpiment, and arsenopyrite samples were obtained from mineral collections at the Department of Geosciences of the Arctic University of Norway in Tromsø and the Mineralogy and Petrology Department, University of Zagreb, Croatia. The realgar and arsenopyrite samples were separated by microdrilling from selected ore samples (Table 2). Before microdrilling, slabs of approximately 1 × 1 × 0.5 cm dimensions were cut from hand specimens containing realgar and arsenopyrite crystals. The slabs were manually polished using 6-, 3- and 1-micron diamond pastes and suitable polishing cloths. After polishing, the realgar and arsenopyrite crystals were checked for the absence of sulfide inclusions by reflected light microscopy (Leica DMJP microscope, Leica Microsystems, Wetzlar, Germany). Optically pure individual grains of orpiment from mineralized samples were handpicked under a binocular microscope and powdered in an agate mortar. The purities of separated arsenic sulfide samples were confirmed by X-ray diffractometry (Philips PW 3040/60 X’Pert PRO powder diffractometer, Malvern Panalytical, Almelo, Netherlands). The studied mercury sulfide samples were obtained from the IDYST-UNIL sample collection. Two samples of pure cinnabar and one mineralized sample (SLO-ID-Cin-171) were from the Idrija mercury deposit (Slovenia) [16]. Two mineralized samples (ES-ALM-Cin-1 and ES-ENT-Cin-1) came from the Almadén mining district (Spain) [32]. The cinnabar from these last three very fine-grained ore samples was preconcentrated by microdrilling with a dental drill and was not purified further.

### Experimental setup

**Fig. 1:**
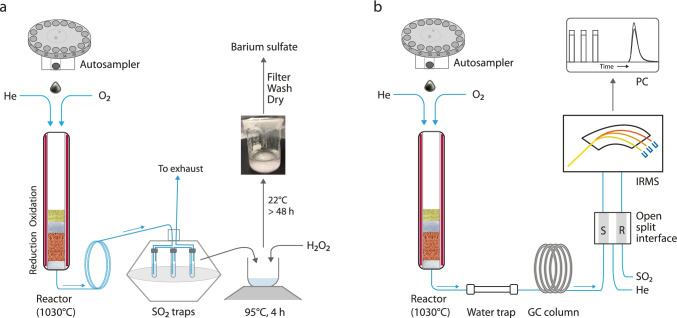
Schematic diagram of the BaSO_4_-EA/IRMS system consisting of an autosampler, a single combined oxidation–reduction reactor, a sulfur dioxide trap, an elemental analyzer, a ConFlo interface, and an isotope ratio mass spectrometer. **a** The sulfur dioxide produced by combustion in the oxidation–reduction reactor is trapped in an aqueous barium chloride solution and oxidized by hydrogen peroxide to form barium sulfate. **b** The sulfur isotope composition of the barium sulfate is measured in an EA/IRMS

An EA combustion/reduction system combined with a trapping system has been developed at the Institute of Earth Surface Dynamics of the University of Lausanne (IDYST-UNIL) to purify and preconcentrate sulfur from various materials before EA/IRMS sulfur isotope analysis (Fig. 1). The combustions were performed in a Carlo Erba 1108 elemental analyzer (Fisons Instruments, Milan, Italy) using the left reactor tube of the furnace packed for sulfur determination and heated at 1030 °C. The inhouse packing of the single combined oxidation–reduction reactor for sulfur isotope analysis is shown in Fig. 1 and detailed in Electronic Supplementary Material, ESM, Fig. S1. In brief, the lower part functioning as a reduction reactor was filled with 30 mm quartz wool, followed by 90 mm of reduced copper wires of 0.7 mm diameter, and a 45 mm layer of quartz chips separating the reduction part from the filling of the upper oxidation part, which consisted of 45 mm WO_3_. The top of the reactor filling did not end with a layer of quartz wool, and no quartz insert was used. With this packing, the reactor was generally useful for combustion of 250–300 sulfide samples and 150–200 sulfate samples. For relatively pure sulfides (i.e., mechanically separated under a binocular microscope and then powdered using mortar and pestle), usually containing 13 to 54 wt.% TS and sulfates with 13 to 24 wt.% TS, depending on their chemistry, the size of sample aliquot ranges from 100 to 1500 μg and 400 to 1500 μg, respectively.

In the off-line EA combustions, the reactor bottom outlet was connected to the trapping system by a 250 mm long polytetrafluoroethylene (PTFE) capillary tube with 1 mm ID and 2 mm OD (Semadeni AG, Ostermundigen, Switzerland). The gaseous products resulting from combustion, with SO_2_ as the main sulfur product, were preconcentrated in a trapping system. Sulfur trioxide may be produced if the reduced copper filling in the lower reduction part of the reactor tube does not reduce it quantitatively to sulfur dioxide. This can happen if the reduced copper wires are almost completely oxidized.

SO_2_ readily dissolves in water and aqueous solutions. Therefore, the SO_2_ trapping system consisted of three glass tubes containing 8 mL of 10% barium chloride solution with an initial pH of 7 arrayed in tandem (Fig. 1a). The trapping tubes were borosilicate glass thread (SVL 15) tubes with 100 mm length, 16 mm diameter, and 1.8 mm wall thickness, and they were fitted with screw caps and 3-mm-thick PTFE faced silicone seals (VWR, Dietikon, Switzerland). Two small holes were carefully perforated at the top of the cap (and seal) to allow 35-cm-long PTFE capillaries (2 mm OD) to pass through very tightly. One capillary was inserted to a depth of up to 5 mm from the tube bottom, and the other capillary was inserted approximately 5–10 mm across the seal. A small amount of all-purpose glue (UHU, Massagno, Switzerland) on the upper external surface of the seal ensured the position and leak-free insertion of the capillary. The absence of leaks was checked periodically with an electronic leak detector (BGB, Böckten, Switzerland). The sulfur oxide trapping system was placed on a porcelain disc (235 mm diameter) within a glass desiccator (VWR, Dietikon, Switzerland). The last PTFE capillary of the trapping tubes was 50 cm long, and it passed through the desiccator lid outlet into the piping of the laboratory extraction system for toxic and hazardous gases (Fig. 1a).

Samples and standards were dried at 50 °C for at least 24 h before analysis. Several aliquots (*n* = 4–6) of the same sample or standard were weighed and wrapped separately in pressed tin capsules for solids (3.3 mm × 5 mm) that were placed in the autosampler (AS-200LS, Fisons Instrument, Milan, Italy) of the elemental analyzer before combustion. Individual aliquots weighed 5000–20,000 µg for arsenides, 200–1000 µg for sulfides (e.g., As_2_S_2,_ As_2_S_3,_ FeAsS, and HgS), and 500–2000 µg for sulfates (e.g., BaSO_4_). To obtain complete (quantitative) oxidation of the sulfur, oxygen (O_2_) injection by the elemental analyzer was optimized (O_2_ closing time 90 s), and vanadium pentoxide (V_2_O_5_) serving as an oxidation catalyst was added to capsules containing the samples and standard reference materials. Various tests with laboratory standards and international reference materials with and without the addition of V_2_O_5_ showed no statistically significant differences in the TS contents and δ^34^S values obtained by EA/IRMS. However, samples with low to very low sulfur concentrations (e.g., arsenides) required large sample aliquots for direct EA/IRMS or several EA-combustion cycles of medium-size aliquots when preconcentrated as barium sulfate in the BaSO_4_-EA/IRMS procedure. For consistency, V_2_O_5_ was added in amounts equal to one to two times the amount of the sample or standard. This procedure ensured that the combustion of sample and standard materials was completed with the same excess O_2_. Therefore, the produced SO_2_ gases had a similar oxygen isotope composition, which improved the reproducibility of the δ^34^S measurements.

The capsules with samples or standards were sequentially dropped into the reactor tube by repetitive manual activation of the elemental analyzer cycle lasting 90 s. The EA was activated every 3–4 min with sequential combustions of a sample or reference material capsule and a blank (no capsule). Two blank (no capsule) combustions followed the last sample/standard-bearing capsule. The combustion products were continuously carried from the reactor bottom outlet, and the PTFE capillary tubing was purged with a slow stream of helium into the trapping tubes. The helium flow rate was maintained throughout the sample combustions at 2–3 bubbles per second (correspond approximately to 10–15 mL/min) and increased to 10–15 bubbles per second (correspond approximately to 40–60 mL/min) after the last EA combustion, after which the system was allowed to purge for approximately 2 min. The final blank combustions and flushing with slightly increased helium flow removed residual gases from the reactor, reactor-outlet connections, and transfer line to the trapping solutions. Complete procedure blanks showed that this cleaning step was sufficient to remove potential traces of residual gases quantitatively, thereby avoiding any contamination and memory effects between samples. After the elemental analyzer cleaning step, the trapping tubes were disconnected, and the solutions were combined in a 100 mL glass beaker. The SO_2_ (with possible traces of SO_3_) produced by the EA combustions remained quantitatively dissolved in the barium chloride solution. The PTFE capillaries were thoroughly cleaned with deionized water, rinsed with MQ water, and dried with compressed air, and new tubes with barium chloride solution were placed into the trapping system for the following sample.

### Conversion of sulfur dioxide to barium sulfate

For the conversion of aqueous sulfur dioxide (SO_2_ (aq)) to SO_3_, hydrogen peroxide (H_2_O_2_) was chosen as the oxidant. The oxidation of S(IV) by H_2_O_2_ has been studied in detail because of global environmental interest in the process. The oxidation of anthropogenic SO_2_ to sulfate by H_2_O_2_ in aqueous aerosol particles (e.g., cloud water, rain drops, and urban aerosols) impacts air quality and the climate and causes human health and ecosystem issues [36]. The fixation of gaseous sulfur dioxide, SO_2_ (g), in the aqueous phase and oxidation by H_2_O_2_ can be summarized by the following reactions:

1 $${\mathrm{SO}}_{2}\left(\mathrm{g}\right)\to {\mathrm{SO}}_{2}\left(\mathrm{aq}\right)$$

2 $${\mathrm{SO}}_{2}\left(\mathrm{aq}\right)+{\mathrm{H}}_{2}\mathrm{O}\to {\mathrm{H}}^{+}+{\mathrm{HSO}}_{3}^{-}$$

3 $${\mathrm{SO}}_{2}\left(\mathrm{aq}\right)+2{\mathrm{OH}}^{-}\to {\mathrm{H}}_{2}\mathrm{O}+{\mathrm{SO}}_{3}^{2-}$$

4 $${\mathrm{HSO}}_{3}^{-}+{\mathrm{H}}_{2}{\mathrm{O}}_{2}\to {\mathrm{SO}}_{4}^{2-}+{\mathrm{H}}^{+}+{\mathrm{H}}_{2}\mathrm{O}$$

In the aqueous trapping solution, the absorbed SO_2_ (g) is present in the form of dissolved SO_2_, HSO_3_^−^, and SO_3_^2−^ (reactions 13, 1–3). The formation of hydrogen sulfite (HSO_3_^−^) from aqueous SO_2_ (3) is fast, and HSO_3_^−^ is a much more abundant and reactive species than SO_3_^2−^ [37]. The oxidation of HSO_3_^−^ by H_2_O_2_ (4) follows a proton-catalyzed pathway comprising three steps (5–7) [37, 38]:

5 $${\mathrm{HSO}}_{3}^{-}+\mathrm{HOOH}\to {\mathrm{HOOSO}}_{2}^{-}+{\mathrm{H}}_{2}\mathrm{O}$$

6 $${\mathrm{HOOSO}}_{2}^{-}+{\mathrm{H}}^{+}\to {\mathrm{HOOSO}}_{2}\mathrm{H}$$

7 $${\mathrm{HOOSO}}_{2}\mathrm{H}\to {\mathrm{SO}}_{4}^{2-}+{2\mathrm{H}}^{+}$$

These reactions are pH- and temperature-dependent. Kinetic studies showed that the oxidation of sulfur (IV) by hydrogen peroxide in the absence of buffer at 15 °C is relatively fast in the 0.7–2.2 pH range [38]. Furthermore, the rate of HSO_3_^−^ oxidation by H_2_O_2_ in aqueous nonbuffered solution increases with temperature [38]. Therefore, the solution containing dissolved SO_2_ and barium chloride was acidified to a pH between 1 and 2 with HCl 10%, and 2 mL of H_2_O_2_ 30% solution was added. The precipitation of nanometer- to micrometer-sized particles of barium sulfate (8) occurred immediately (Fig. 1a).

8 $${\mathrm{Ba}}^{2+}+{\mathrm{SO}}_{4}^{2-}\to {\mathrm{BaSO}}_{4}\left(\mathrm{s}\right)$$

The high Ba^2+^ concentration triggered the precipitation of BaSO_4_ and complete oxidation of HSO_3_^−^, even if it was present at low concentration. The sample beaker was covered with a PTFE plate and heated on a hot plate at 95 °C for 4 h with periodic mixing. During this heating at a sub-boiling temperature, the white barium sulfate precipitate was observed to form from amorphous solids, likely corresponding to nanoparticles fusing into micron-sized crystals [39]. The solution was allowed to cool down and kept at room temperature for three days. The precipitated barium sulfate was recovered by passing the solution through a membrane filter with 0.20 µm pore size (regenerated cellulose filter, 47 mm diameter, Sartorius Stedim Biotech, Göttingen, Germany). The filter was washed several times with hot (~ 60–80 °C) MQ water and dried at 40 °C for > 48 h. The recovered barium sulfate was quantified gravimetrically, mixed well to ensure chemical (and isotopic) homogeneity at the few 100 μg to mg scale, and then stored in a desiccator for sulfur isotope analysis by EA/IRMS.

### Sulfur isotope analysis

The sulfur isotopes and the total sulfur content were measured at the IDYST-UNIL laboratories using a Carlo Erba 1108 elemental analyzer connected to a Thermo Fisher (Bremen, Germany) Delta V Plus isotope ratio mass spectrometer that was operated in the continuous helium flow mode via a Conflo III split interface (Fig. 1b) [32]. The temperatures of the elemental analyzer reactor (packed as described earlier) and the gas chromatography (GC) oven were 1030 °C and 80 °C, respectively. The rate of carrier He through the reactor was set at 80 mL/min, and the rate of O_2_ flow was 30 mL/min. The combustion gases were dried by passing them through a glass tube (110 mm long, 10 mm ID, 12 mm OD) filled with 4 g of Mg(ClO_4_)_2_ flakes and then carried through a sulfur separation column (80 cm, 4 mm ID, 6 mm OG, PTFE) packed with Hayesep Q 80–100 mesh (Säntis Analytical, Teufen, Switzerland) for separation of gas components. The SO_2_ in the open split of the Conflo III was directed to the ion source of the IRMS for analysis of sulfur stable isotopes. The stable isotope composition of sulfur was reported in the delta (δ) notation as variation in the molar ratio of the heavy to light isotope [40] of sulfur (^34^S/^32^S) relative to the Vienna Cañon Diablo Troilite (VCDT) standard:

$${\updelta }^{34}\mathrm{S}={\left({}^{34}\mathrm{S}/{}^{32}\mathrm{S}\right)}_{\mathrm{sample}}/{\left({}^{34}\mathrm{S}/{}^{32}\mathrm{S}\right)}_{\mathrm{standard}}-1.$$

We used the Urey unit (Ur) for the delta values, as recommended by the International Union of Pure and Applied Chemistry (IUPAC). One milliUrey (mUr) is equivalent to one per mil (‰); although the ‰ is not an SI unit and is deprecated [41], it remains in use. The SO_2_ standard gas was calibrated against the VCDT scale using the reference material (RM) IAEA-S-1 silver sulfide (Ag_2_S) standard reference material (RM) with a δ^34^S value of − 0.3 mUr. No correction was applied for the contribution of ^18^O/^16^O to ^34^S/^32^S, because the samples and reference materials produced SO_2_ in the same combustion environment with identical excesses and sources of oxygen (i.e., O_2_ and V_2_O_5_). The δ^34^S values were determined by using automatic peak integration (Isodat 3.0 Software, Thermo Fisher Scientific, Bremen, Germany). The normalization of the sample raw δ^34^S values (in mUr) to the VCDT scale was performed with a three-point calibration based on measurements of two sets of three RMs at the beginning and at the end of each analytical sequence. All standards and samples were analyzed in duplicate. For calibration/normalization of the measured δ^34^S values of sulfate samples, the barite RMs NBS 127, IAEA-SO-5, and IAEA-SO-6 were used, and for sulfides, the silver sulfide RMs IAEA-S-1, IAEA-S-2, and IAEA-S3 were used (Table 1). The overall analytical reproducibility of the direct EA/IRMS and BaSO_4_-EA/IRMS analyses was assessed by replicate analyses of laboratory standards (UVA-sulfate, UNIL-Fx-sulfate, UNIL-PyE, UNIL-cinnabar) and RMs (IAEA-S-4 Soufre de Lacq, sphalerite NBS 122 and NBS 123) (Table 1). The δ^34^S values of the RMs are from Brand et al. [42]. The δ^34^S values of the laboratory standards in Table 1 are those obtained during calibrations performed before the start of the BaSO_4_-EA/IRMS experiments (December 2019–January 2020). The obtained δ^34^S values differ slightly (~ +0.3 mUr) from those reported in preceding studies (e.g., 12.5 mUr for UVA barium sulfate, −7.0 mUr for PyE pyrite, and + 12.5 mUr for synthetic cinnabar) [16, 43–45]. These differences arose because the recommended values of the international sulfur isotope reference materials changed slightly (relative to the VCDT scale defined by assigning the value of −0.3 mUr to the silver sulfide primary RM IAEA-S-1) [42]. The barium sulfate recovered from the multiple combustions of sample capsules allowed the replicate sulfur isotope analysis (*n* = 3–4). The reproducibility of the EA/IRMS analyses was better than ± 0.3 mUr (one standard deviation, SD). The accuracy of the δ^34^S analyses was checked periodically by analyses of RMs. The total sulfur content (wt.% TS) was determined from the sum of the peak areas of the major isotopes (*m*/*z* 64 and 66). This integrated peak area was calibrated to TS concentration by using different aliquot sizes of the same standards used for normalization of the δ^34^S values.

## Results and discussion

The δ^34^S and TS values obtained by direct EA/IRMS and by preconcentration as barium sulfate before EA/IRMS (BaSO_4_-EA/IRMS) are presented in Tables 3–5 and ESM Tables S1–S3. The presentation and discussion of the results are divided into two sections titled “Evaluation of the analytical procedure using reference materials” and “Application to analyses of arsenides and arsenic and mercury sulfides.”

**Table 3:** Total sulfur content of the sulfur isotope reference materials and laboratory standards obtained by direct EA/IRMS and by BaSO_4_-EA/IRMS

			Direct EA/IRMS					BaSO_4_-EA/IRMS				
Identifier	Material	Theor. *TS*^a^ (wt.%)	*TS* (wt.%)	*SD*^b^	2 *SE*^b^	*n*	Recovery (%)^c^	*TS* (wt.%)	*SD*^b^	2 *SE*^b^	*m*	Recovery (%)^c^
NBS 127	Barite	13.74	13.43	0.71	0.37	14	97.7	13.26	1.60	1.56	4	96.5
IAEA-S-4	Sulfur	100.00	98.95	1.03	0.71	8	98.9	96.24	2.12	2.94	4	96.2
NBS 122	Sphalerite	32.83	29.43	1.39	0.86	10	89.6	27.55	4.17	5.78	4	83.9
NBS 123	Sphalerite	32.83	32.50	0.85	0.53	10	99.0	31.37	2.08	2.88	4	95.6
UVA-sulfate	BaSO_4_	13.74	13.86	0.47	0.24	15	100.9	13.17	0.95	0.94	4	95.8
Fx-sulfate	BaSO_4_	13.74	13.51	0.83	0.52	10	98.3	12.53	0.88	0.77	4	91.2
UNIL-PyE ^d^	Pyrite	53.45	37.04	1.31	0.70	14	-	36.61	2.33	1.86	4	-
UNIL-Cinnabar	Cinnabar	13.78	13.79	0.30	0.17	12	100.1	13.49	0.78	0.83	4	97.9

^a^Theoretical total sulfur content in wt.% determined from the stoichiometry

^b^Mean and standard deviation (*SD*) of *n* replicate analyses or *m* independent experiments; 2 *SE* denotes two standard errors of the mean (95% CI)

^c^Percent recovery calculated from the TS obtained by direct EA/IRMS or by gravimetric quantification of the barium sulfate

^d^Oxidative overgrowth on the pyrite grains, checked by XRD and under the microscope, explain the lower *TS* wt.% value. The sulfur isotope composition of this inhouse-standard remained constant (within analytical uncertainty) for the last 25 years

### Evaluation of the analytical procedure using reference materials

For evaluation of the developed analytical procedure (BaSO_4_-EA/IMS), blank analysis, total sulfur recovery, and the accuracy and precision of sulfur isotope analyses of RMs and laboratory sulfide and sulfate standards were considered. The δ^34^S and TS values were reported by using the mean for replicate analyses, the standard deviation (SD), and the standard error of the mean (SE).

The uncertainties reported in Tables 3, 4, and 5 correspond to intermediate precisions, including repeatability and reproducibility, and were estimated from the SDs and SEs of more than four independent experiments/analyses performed during six analytical sessions between December 2020 and May 2021. The SD was used to show how widely scattered the measurements were. The SE values indicated the uncertainty of the mean measurement (i.e., precision) and were calculated as SE = SD/$\surd$ *n*, where *n* is the number of individual analyses. The SE values were multiplied by 1.96 to give the 95% confidence interval for the mean and reported in tables as 2 SE for simplicity. The accuracy of the procedures was assessed by the closeness of agreement (i.e., the difference) between the mean value obtained from analytical sessions and an accepted (i.e., recommended) reference value.

**Table 4:** Sulfur isotope composition of the sulfur isotope reference materials and laboratory standards obtained by direct EA/IRMS and by BaSO_4_-EA/IRMS

			Direct EA/IRMS					BaSO_4_-EA/IRMS			
Identifier	Material	Recom. δ^34^S^a^ (mUr or ‰)		δ^34^S_VCDT_ (mUr or ‰)	SD^b^	2 SE^b^	*n*	δ^34^S_VCDT_ (mUr or ‰)	SD^b^	2 SE^b^	*m*
NBS 127	Barite	21.12		21.05	0.38	0.20	14	20.98	0.43	0.42	4
IAEA-S-4	Sulfur	16.9		17.09	0.18	0.12	8	16.62	0.27	0.27	4
NBS 122	Sphalerite	0.18		−0.01	0.17	0.11	10	0.31	0.11	0.11	4
NBS 123	Sphalerite	17.09		17.22	0.22	0.14	10	17.08	0.29	0.29	4
UVA-sulfate	Barium sulfate	12.71		12.66	0.23	0.12	15	12.99	0.34	0.28	6
Fx-sulfate	Barium sulfate	17.90		17.86	0.26	0.16	10	18.02	0.25	0.24	4
UNIL-PyE	Pyrite	−6.72		−6.69	0.20	0.09	18	−6.74	0.43	0.34	6
UNIL-Cinnabar	Mercury sulfide	15.82		15.79	0.14	0.08	11	15.67	0.25	0.24	4

^a^Recommended δ^34^S value of the international reference materials and laboratory standards in Table 1

^b^Mean and standard deviation (*SD*) of *n* replicate analyses or *m* independent experiments; 2 *SE* denotes two standard errors of the mean (95% CI)

**Table 5:** Total sulfur content and sulfur isotope composition of arsenide and arsenic and mercury sulfides obtained by direct EA/IRMS and by BaSO_4_-EA/IRMS

	Direct EA/IRMS							BaSO_4_-EA/IRMS						
Lab code^a^	TS (wt.%)	SD	2 SE	δ^34^S_VCDT_ (mUr or ‰)	SD^b^	2 SE^b^	*n*	TS (wt.%)	SD	2 SE	δ^34^S_VCDT_ (mUr or ‰)	SD^b^	2 SE^b^	*m*
AR-1								1.62	0.31	0.30	–8.31	0.19	0.18	4
AR-2								2.21	0.36	0.49	–7.63	0.14	0.20	2
AR-3								1.16	0.27	0.37	–5.21	0.07	0.10	2
AR-4								0.32	0.11	0.15	4.91	0.28	0.39	2
AR-5								0.64	0.33	0.46	1.00	0.13	0.18	2
Rlg-1	30.05	0.79	1.09	–3.31	0.15	0.20	2	28.98	0.85	1.18	–2.95	0.33	0.46	2
Rlg-2	32.56	0.55	0.76	–4.43	0.09	0.12	2	30.52	1.39	1.76	–4.37	0.21	0.28	2
Rlg-3	31.75	0.42	0.58	3.75	0.08	0.11	2	28.21	0.94	1.31	3.55	0.18	0.25	2
Orp-1	33.72	0.19	0.26	–1.30	0.47	0.66	2	30.54	1.14	1.58	–1.51	0.15	0.21	2
Orp-2	36.88	1.03	1.43	–1.71	0.29	0.41	2	34.72	2.59	3.59	–1.63	0.28	0.37	2
Orp-3	35.58	1.43	1.99	–1.13	0.20	0.28	2	35.74			–1.18			1
Orp-4	36.06	1.49	2.06	1.25	0.12	0.16	2	35.36	2.18	3.02	1.06	0.17	0.24	2
Apy-1	16.16	0.63	0.62	6.02	0.05	0.05	4	15.19	0.95	1.32	5.87	0.34	0.47	2
Apy-2	16.54	0.16	0.16	6.00	0.10	0.09	4	16.57	1.08	1.46	6.11	0.27	0.38	2
Apy-3	16.33	0.31	0.31	–0.42	0.05	0.05	4	15.33	1.36	1.88	–0.68	0.22	0.30	2
Apy-4	20.90	0.10	0.10	4.16	0.22	0.21	4	19.46	1.74	2.41	4.11	0.29	0.40	2
Apy-5	16.03	0.28	0.27	4.07	0.13	0.13	4	15.87	1.86	2.57	4.34	0.25	0.35	2
Cin-1	13.67	0.13	0.10	–2.45	0.16	0.13	6	13.18	1.05	1.46	–2.21	0.20	0.28	5
Cin-2	13.65	0.17	0.14	–1.34	0.11	0.09	6	12.83	0.93	1.28	–1.15	0.30	0.41	3
Cin-3	9.51	0.24	0.33	–4.13	0.14	0.19	2	9.89	1.13	1.56	–3.72	0.21	0.29	2
Cin-4	13.72	0.20	0.28	4.93	0.21	0.29	2	13.01	1.51	2.09	4.73	0.17	0.23	2
Cin-5	6.85	0.14	0.19	8.56	0.13	0.17	2	6.93	0.17	0.24	8.30	0.22	0.30	2

^a^ Mineralogy and origin of the sulfide samples are presented in Table 2. AR = arsenide; Rlg = realgar; Orp = orpiment; Apy = arsenopyrite; Cin = cinnabar

^b^ Mean and standard deviation (*SD*) of *n* replicate analyses or *m* independent experiments; 2 *SE* denotes two standard errors of the mean (95% CI)

#### BaSO_4_-EA/IRMS and EA/IRMS blanks

All the blanks of the matrix separation/sulfur preconcentration step showed no detectable BaSO_4_ precipitates when the effluent of eight blank combustions (six with capsules containing V_2_O_5_ and two with no capsule) was trapped. Therefore, there was no appreciable memory effect or carry-over of SO_2_ from previous samples, indicating that the EA reactor tube and the transfer PTFE capillaries, when purged with the final blank combustions and a slight increase of the helium carrier flow, quantitatively removed potential residual gases. The EA/IRMS analytical sequence blanks for sulfates and sulfides showed no detectable SO_2_ peaks at m/z 64 and 66. Rarely was it possible to observe a slight increase in the background signals. This potential memory effect had little impact on the average δ^34^S and TS values of the replicate IRMS measurements. The increase in background signals was restricted by conditioning the sulfur separation GC column at 95 °C for at least 2 h.

#### Total sulfur content and percent recovery

**Fig. 2:**
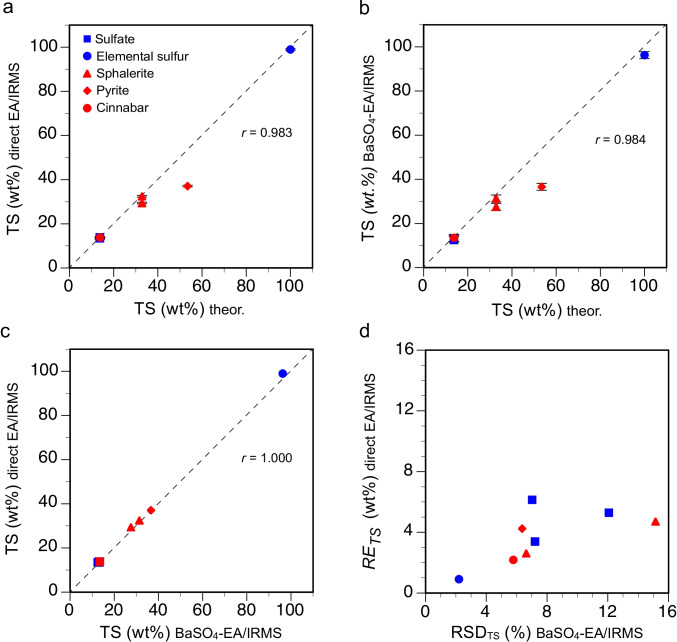
Total sulfur (TS) content in reference materials and laboratory standards from direct EA/IRMS (**a**) and BaSO_4_-EA/IRMS (**b**) compared with the theoretical values determined from the stoichiometry and compared together (**c**). **d** Relative standard deviations (RSDs) for both methods. The error bars represent two standard errors of the mean (2 SE) from four or more replicate analyses (see Table 3). The dashed line is the 1:1 line

The eight RMs and laboratory standards used for validation of the developed BaSO_4_-EA/IRMS procedure covered a broad range of TS values (13.74 to 100.00 wt.%) defined by the barite and elemental sulfur standards (Table 3, ESM Table 1). The TS values determined from the integrated peak areas of the major isotopes from direct EA/IRMS and those determined gravimetrically in the BaSO_4_-EA/IRMS procedure were highly correlated with one another and with the theoretical TS determined from the stoichiometry (the Pearson correlation coefficients *r* were 1.000, 0.983, and 0.984, respectively, *n* = 8, *p* < 0.001) (Figs. 2a, b, c). In particular, the relative difference of the TS values from direct EA/IRMS with the theoretical values ranged between −0.9 and 2.3%; the TS values from BaSO_4_-EA/IRMS differed from the theoretical values by 2.1 to 8.8%. The direct EA/IRMS values agreed with the theoretical values to within a range of −0.9 to 2.3%; the BaSO_4_-EA/IRMS values agreed with the theoretical values to within a range of 2.1 to 8.8%. The higher offsets were observed for the laboratory standard PyE, which showed evidence of alteration, as indicated by oxidation halos around the pyrite grains that were only visible under the microscope, and for NBS 122, which is known to be discontinuous and possibly nonhomogeneous [42]. The reproducibility and precision of TS analyses by direct EA/IRMS (SD: 0.86 ± 0.38 wt.% and 2 SE: 0.51 ± 0.24 wt.%, with *n* = 8 to 15) were better than those for BaSO_4_-EA/IRMS (SD: 1.86 ± 1.11 wt.% and 2 SE: 2.20 ± 1.68 wt.%, *n* = 4 for all standards). The relative standard deviation (RSD = 1 SD/mean × 100), another estimate used for the comparison of the intermediate precision of the TS analyses, was lower for direct EA/RMS (3.7 ± 1.7%) than for BaSO_4_-EA/IRMS (7.8 ± 4.0%) (Fig. 2d). Notably, the BaSO_4_-EA/IRMS method showed large RSDs (> 10%) for NBS 127 and NBS 122, which were most likely associated with incomplete recovery of the BaSO_4_ from some filters (*n* = 14 for NBS 127) and sample heterogeneity (for NBS 122).

The percent recovery (*R*), calculated from the TS obtained by direct EA/IRMS and the theoretical TS, ranged from 97.7 to 100.9% (99.2 ± 1.1%) when NBS-122 and PyE were excluded (Table 3). For the BaSO_4_-EA/IRMS procedure, the percent recovery varied between 91.2 and 97.9% (95.5 ± 2.3%). The lower *R* values for the BaSO_4_-EA/IRMS procedure compared with those for direct EA/IRMS may be explained by (i) some potential loss of BaSO_4_ when handling and filtering the SO_2_ trapping solutions, (ii) incomplete recovery of the solid BaSO_4_ from the dried filters, and (iii) uncertainties in the sum of the weights of the combusted aliquots and the weight measurements of recovered BaSO_4_.

#### Comparison of the δ^34^S values from direct EA/IRMS and BaSO_4_-EA/IRMS

**Fig. 3:**
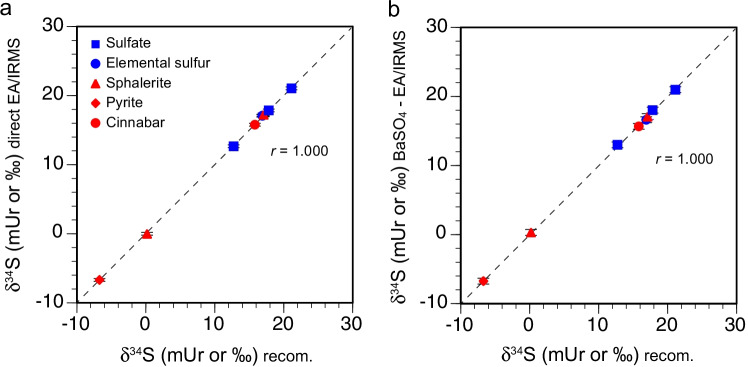
Sulfur isotope ratios (δ^34^S) in reference materials and laboratory standards from direct EA/IRMS (**a**) and BaSO_4_-EA/IRMS (**b**) compared with the recommended values. Error bars represent two standard errors of the mean (2 SE) from four or more replicate analyses (see Table 4). The dashed line is the 1:1 line

**Fig. 4:**
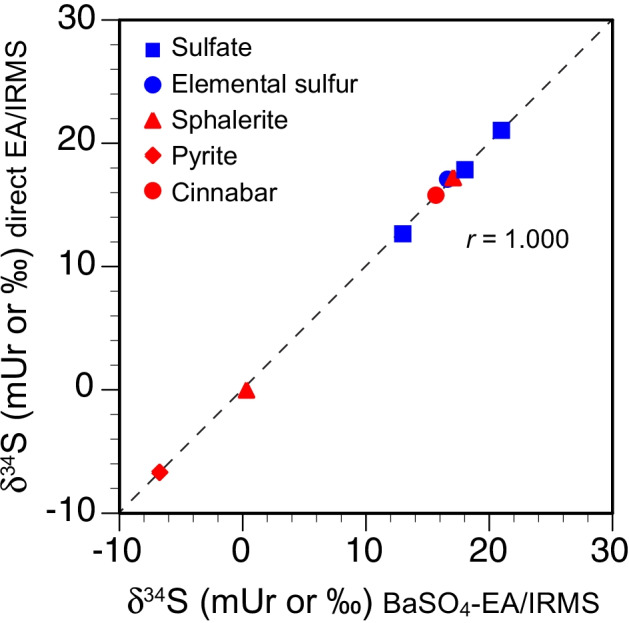
Comparison of the δ^34^S values in reference materials and laboratory standards from direct EA/IRMS and BaSO_4_-EA/IRMS. The dashed line is the 1:1 line

The δ^34^S values of the RMs and laboratory standards covered a wide range from −6.72 to + 21.12 mUr (Table 4, ESM Table 2). The δ^34^S values determined by direct EA/IRMS and those determined via the BaSO_4_-EA/IRMS procedure were highly correlated with the accepted/recommended values (Figs. 3a, b) and with each other (Fig. 4). The average SD and 2 SE values for direct EA/IRMS (0.22 ± 0.07 mUr and 0.13 ± 0.04 mUr, respectively) did not differ significantly (*p* > 0.001) from those obtained by BaSO_4_-EA/IRMS (0.30 ± 0.11 mUr and 0.27 ± 0.09 mUr). The long-term reproducibility (i.e., intersession repeatability), estimated from variability of the 2 SE values, was lower than 0.14 and 0.20 mUr for direct EA/IRMS of sulfides and sulfates, respectively, and lower than 0.34 and 0.42 mUr for BaSO_4_-EA/IRMS of sulfides and sulfates, respectively (Table 4).

The accuracies were assessed by the agreement between the mean value obtained from RMs and laboratory standards and the recommended or accepted δ^34^S values for both procedures. The difference between the BaSO_4_-EA/IRMS and the accepted δ^34^S values (0.00 ± 0.12 mUr) was similar to those for direct EA/IRMS (0.00 ± 0.19 mUr). These comparable precision and accuracy for both procedures are not surprising, as both achieved high total sulfur recoveries (generally > 95%).

In summary, the high correlations of the TS values obtained by direct EA/IRMS and by BaSO_4_-EA/IRMS with the theoretical TS values and the high recoveries indicate that for pure sulfur compounds or minerals (RMs and laboratory standards), the combustion of sulfur in the optimized elemental analyzer conditions was complete, as the produced SO_2_ was trapped in the form of BaSO_4_. The good precision and the closeness of agreement between the mean δ^34^S values obtained from replicate BaSO_4_-EA/IRMS analyses and the recommended reference values indicate that the BaSO_4_-EA/IRMS procedure does not introduce isotope fractionation, despite the numerous EA and wet chemistry steps required for the preparation of the BaSO_4_ analyte for EA/IRMS measurement. This lack of procedure-induced isotope fractionation validates the suitability of the proposed BaSO_4_-EA/IRMS procedure for sulfur isotope analyses of geological and environmental materials with low sulfur content and challenging matrices.

### Application to analyses of arsenides and arsenic and mercury sulfides

**Fig. 5:**
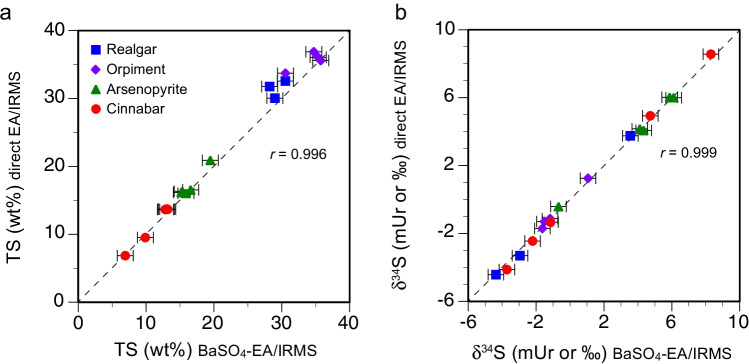
Comparison of the TS contents (**a**) and δ^34^S values (**b**) in arsenic and mercury sulfides from direct EA/IRMS and BaSO_4_-EA/IRMS. Error bars represent two standard errors of the mean (2 SE) from two or more replicate analyses (see Table 5). The dashed line is the 1:1 line

The applicability of the proposed BaSO_4_-EA/IRMS procedure was evaluated by analyses of sulfur isotopes and TS contents in arsenides (*n* = 5), arsenic sulfides (realgar and orpiment, *n* = 3 and 4, respectively), sulfarsenide (arsenopyrite, *n* = 3), and mercury sulfide (cinnabar and cinnabar ore, *n* = 2 and 3, respectively) (Table 5, ESM Table 3). The sulfarsenide and sulfide samples, but not the arsenides, were also analyzed by direct EA/IRMS. The TS contents of the arsenides determined by BaSO_4_-EA/IRMS varied between 0.32 and 2.21 wt.%, within a similar range as the in situ values obtained by microprobe analyses (0.83 to 3.03% S; [35]). The TS contents of the arsenic and mercury sulfide separates ranged between 13.65 and 36.88 wt.%, excluding the cinnabar ore samples Cin-3 and Cin-5) with TS values 9.51 and 6.85 wt.%, respectively. For the arsenides, the RSD values were high (28.93 ± 14.40%), which may be explained by the low sulfur content and heterogeneous distributions of sulfur in the minerals (Table 5, ESM Table 3). Similar high RSDs were observed for TS levels determined by EA/IRMS of low sulfur shale samples [46]. The TS values of the pure sulfide separates and ore samples determined by BaSO_4_-EA/IRMS (6.93–35.74 wt.%) were highly correlated (*r* = 0.996, *p* < 0.001) with the values obtained by EA/IRMS (6.85–36.88 wt.%) (Fig. 5a). However, the long-term reproducibility of the TS analyses by BaSO_4_-EA/IRMS (RSD = 6.78 ± 3.26%) was poorer by a factor of approximately 2–3 compared to that for direct EA/IRMS (RSD = 2.02 ± 1.16%).

The δ^34^S values from BaSO_4_-EA/IRMS of the arsenides varied between − 8.31 and + 4.91 mUr, and those for sulfides varied between − 4.37 and + 8.30 mUr (Table 5, ESM Table 3). The δ^34^S values obtained by BaSO_4_-EA/IRMS for arsenopyrite, arsenic, and mercury sulfides were highly correlated with the EA/IRMS values (*r* = 0.999, *p* < 0.001) (Fig. 5b). As with the RMs and laboratory standards, the precision for analyses of sulfur isotope ratios of arsenic and mercury sulfides was similar when using BaSO_4_-EA/IRMS (2 SE = 0.32 ± 0.09) or EA/IRMS (2 SE = 0.20 ± 0.15).

#### Quantitative sulfur combustion and potential release of toxic gases

The completeness of the sample sulfur combustion when using the optimized EA conditions in pure sulfides and sulfates was proven by comparing the TS values obtained by EA/IRMS or BaSO_4_-EA/IRMS and the theoretical for pure sulfur compounds used as RMs and laboratory standards (Table 3). A final experiment was performed to investigate the effect of low sulfur concentrations and complex matrices on the completeness of the sulfur combustion as well as the potential release from the elemental analyzer reactor of gases containing arsenic or mercury compounds. Four tin capsules with known weights of sample, summing to a total between 20 and 35 mg, were prepared for an arsenide (Ar-1, nickeline, NiAs), orpiment (Orp-3), and cinnabar (UNIL-Cinnabar standard). The sample capsules were combusted as described in the matrix-separation/preconcentration step. The released gases were collected in three sequential trapping tubes (A, B, and C) arrayed in tandem. Each tube contained 8 mL MQ water. The concentrations of sulfur, arsenic, and mercury in the solutions of the trapping tubes A, B, and C were determined by inductively coupled plasma optical emission spectrometry (ICP-OES; Agilent 5900 SVD; Agilent Technologies Inc., Santa Clara, CA, USA) calibrated to certified standards. The accuracy and precision of the ICP-OES measurements were checked using standard solutions. No 10% barium chloride solution was used as trapping solution to avoid potential matrix interferences and high background in the ICP-OES measurements. Each sample was done in replicate.

The sulfur was completely retained in the first trapping tube (A) solution for all samples; only trace amounts were measured in tubes B and C (less than 0.08% of the concentration in tube A). The TS calculated from the mg/L concentrations in the trapping solutions and the total weight of combusted sample aliquots were 1.82 ± 0.20 wt.% for the arsenide sample AR-1, 35.47 ± 0.77 wt.% for the orpiment sample Orp-3, and 13.78 ± 0.11 wt.% for UNIL-cinnabar. These values are well within the uncertainty with the TS values determined gravimetrically from the barium sulfate obtained y BaSO_4_-EA/IRMS (Table 5). This good match of the TS results indicates that the sulfur was completely combusted during the repeated analytical cycles of the elemental analyzer with optimized oxidation conditions and SO_2_ collection in the trapping solutions. Arsenic was not detected in any of the trapping solutions. The only explanation for this finding is that arsenic remained in the reactor quartz tube after heating at a temperature of 1030 °C. Most likely, the arsenic oxidation products reacted with V_2_O_5_, WO_3_, Cu, and their reduction/oxidation products to form vanadium-copper-tungsten-arsenate oxides (e.g., (VO_3_)_2_(AsO_3_)_2_, (VO_2_)_2_AsO_4_, W_3_(AsO_4_)_4_, Cu_3_(AsO_4_)_2_). This is important because EA/IRMS analyses of arsenopyrite and other sulfarsenides and arsenic-rich sulfides may not cause contamination of the analytical instrument or be a source of arsenic exposure in the work place.

For the cinnabar sample, all the trapping tube solutions contained between 15.7 and 108.4 µg/L mercury, decreasing from the first trapping tube (100.0 ± 11.8) to the third (23.5 ± 11.1). The mercury retained in the solutions corresponded to 0.01 to 0.04% of the total mercury in the combusted cinnabar samples. These results provide direct evidence that a trace amount of mercury gases is released from the reactor tube of an elemental analyzer for sulfur isotope analysis (filled with WO_3_, quartz chips, Cu wire, and quartz wool). In the EA/IRMS system the mercury released by decomposition of HgS, HgO, or mercury-containing compounds will be most likely be retained in cooler parts (< 320 °C), mostly by metal oxides [23, 47] within the EA system, including the connections at the bottom outlet of the reactor, the connecting capillaries, and the packings of the water trap and GC column. Sulfarsenides (e.g., arsenopyrite) and arsenic and mercury sulfides can be analyzed by direct EA/IRMS with acceptable precision and accuracy. However, an analytical problem remains due to the lack of composition-matched standard RMs for the use in calibration of the measured δ^34^S values, which may be more problematic when analyzing low sulfur-containing arsenides.

#### Sample throughput for BaSO_4_-EA/IRMS

The proposed procedure was primarily developed for the safe, accurate, and precise sulfur isotope analysis via EA/IRMS of low sulfur-containing arsenides carefully separated from complex ore samples. The BaSO_4_ formation is a necessary wet-chemistry step allowing the matrix separation and sulfur-preconcentration, carefully considering the HES issues. The wet-chemistry step preceding the sulfur isotope analysis by EA/IRMS is relatively time-consuming. However, it compares relatively favorably with similar wet-chemistry steps required to prepare samples for sulfur isotope analysis, such as the extraction of carbonate-associated sulfate (CAS) [48, 49]. The steps of SO_4_^2−^ precipitation with BaCl_2_, filtration, drying and recovery of the BaSO_4_, and δ^34^S measurement by EA/IRMS are similar, but the wet-chemistry stages involving sample washing, acid treatments, and filtration are replaced in the BaSO_4_-EA/IRMS procedure by the collection of the gaseous products from consecutive elemental analyzer combustions in a trapping solution, while substantially shortening the preparation time. The first preparation step, including EA combustions (e.g., ten analytical cycles, including sample aliquots and blanks), purging the system, and changing trapping tubes, restricts the sample preparation throughput to 14 to 18 samples per day. The EA/IRMS system allows the δ^34^S measurement of up to 100 samples per day.

## Conclusions

A method was developed for accurate and precise sulfur isotope analysis in samples of low to very low sulfur concentration and samples with a complicated matrix, which may release toxic gases upon combustion in the elemental analyzer, with risk of instrument contamination and health effects in the working environment. Multiple sample aliquots are sequentially combusted in an elemental analyzer, and the SO_2_ produced is trapped in a barium chloride solution. Quantitative oxidation of SO_2_ by H_2_O_2_ produces a homogeneous precipitate of BaSO_4_, which is subjected to sulfur isotope analysis by direct EA/IRMS with international reference sulfate materials used for calibration. The long-term reproducibility and accuracy of δ^34^S values and total sulfur contents are similar to those achievable by direct EA/IRMS of sulfide and sulfate samples. These equally accurate and precise data indicate that the matrix-separation/sulfur-preconcentration step before EA/IRMS does not cause sulfur isotope fractionations.

Additionally, the δ^34^S values for arsenic and mercury sulfides obtained by BaSO_4_-EA/IRMS are comparable with those from direct EA/IRMS. Arsenic was not detected in any of the trapping solutions, providing direct evidence that direct sulfur isotope analysis of arsenopyrite, other sulfarsenides, and arsenic-rich sulfides by EA/IRMS does not cause arsenic contamination of the instrument and is not a source of arsenic exposure in the working environment. Trace levels of mercury were detected in the trapping solution when combusting cinnabar (i.e., < 0.08% of the combusted mercury amount). The released volatile mercury compounds will probably be retained in cooler parts within the EA system, including the connections at the bottom outlet of the reactor, the connecting capillaries, and the packings of the water trap and GC column.

Finally, the wet-chemistry step of matrix separation and sulfur preconcentration in the BaSO_4_-EA/IRMS procedure avoid potential problems associated with direct EA/IRMS: (a) incomplete combustion of sulfur in large samples, (b) heterogeneity of materials with low sulfur content, and (c) memory effects and risk of instrument contamination in challenging complex matrices. Additionally, the δ^34^S measurement of the derived barium sulfate samples can be normalized and validated with well-constrained matrix-matched international reference materials with certified sulfur isotope ratios. The relatively low sample throughput, of less than 18 samples per day, in the BaSO_4_ preparation step, is well balanced concerning analytical, instrumental, health, safety, and environmental benefits. The developed method allows the accurate and precise sulfur isotope measurement at low to very low sulfur concentrations and in challenging matrices, including arsenides, rocks, sediments, soils, fossil, and recent biological material.

## Supplementary Information

Below is the link to the electronic supplementary material.

Supplementary file1 (PDF 766 KB)

Supplementary file2 (XLSX 16 KB)

Supplementary file3 (XLSX 14 KB)

Supplementary file4 (XLSX 17 KB)

## Data Availability

All the data are included and described within the manuscript and supplementary material.
