# Supplementary material for: Safe, accurate, and precise sulfur isotope analyses of arsenides, sulfarsenides, and arsenic and mercury sulfides by conversion to barium sulfate before EA/IRMS
Source: Anal Bioanal Chem. 2022 Jan 23;414(6):2163–79. doi: 10.1007/s00216-021-03854-y (PMC8821489; doi:10.1007/s00216-021-03854-y)
Supplement: Supplementary file 1 — Supplementary file1 (PDF 766 KB) [file 216_2021_3854_MOESM1_ESM.pdf]

Analytical and Bioanalytical Chemistry

Electronic Supplementary Material:

Safe, accurate, and precise sulfur isotope analyses of arsenides, sulfarsenides, and arsenic and mercury sulfides by conversion to barium sulfate before EA/IRMS

Jorge E. Spangenberg [1,*], Nicolas J. Saintilan [2], Sabina Strmić Palinkaš [3]

[1] *Institute of Earth Surface Dynamics (IDYST), University of Lausanne, CH-1015 Lausanne, Switzerland*

[2] *Institute of Geochemistry and Petrology, Department of Earth Sciences, ETH, CH-8092 Zürich, Switzerland*

[3] *Department of Geosciences, UiT The Arctic University of Norway in Tromsø, N-9037 Tromsø, Norway*

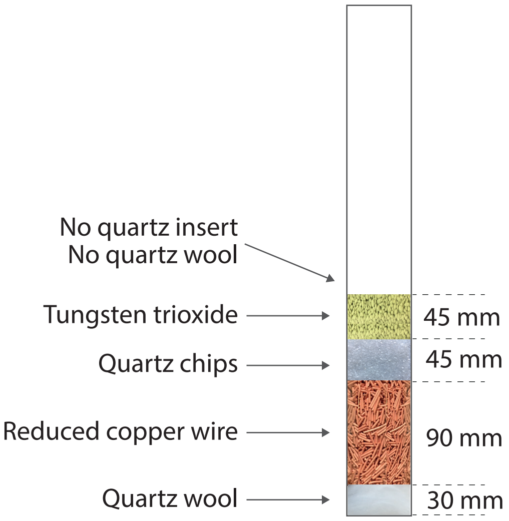

Fig. S1 Packing of reactor for sulfur isotope analysis by EA/IRMS.
